# Mindfulness and compassion training for health professionals: A qualitative study

**DOI:** 10.3389/fpsyg.2022.1113453

**Published:** 2023-01-12

**Authors:** Clémence Brun, Alexis Akinyemi, Laurène Houtin, Claire Mizzi, Thierry Cardoso, Corinne Isnard Bagnis

**Affiliations:** ^1^Université Grenoble Alpes, TIMC-IMAG UMR CNRS, ThEMAS Team, La Tronche, France; ^2^Laboratoire Parisien de Psychologie Sociale, Nanterre, France; ^3^APHP Sorbonne University, Paris, France; ^4^Santé Publique France, Saint-Maurice, France

**Keywords:** compassion, stress, burnout, patient-centered care, mindfulness

## Abstract

**Background:**

Compassion is a key component of quality care. Encouraging Health Care Professionals (HCPs) to develop a patient-centered care relationship through mindfulness and compassion training may be beneficial for both patients and HCPs.

**Method:**

We assessed the impact of a compassion-centered mindfulness program [i.e., the Mindfulness Based (MB) CARE program] on healthcare practice conducting 10 phone interviews with HCPs who experienced the program.

**Results:**

The training had an overall positive impact on the HCPs ability to feel compassion toward their patients and themselves, helped them develop kindness toward themselves and their patients, and enhanced their attention to their patient’s needs and theirs. Participants were better able to accept the difficult work experiences or those their patients experienced, with more perceived equanimity and less reactivity.

**Conclusion:**

Professional mindfulness and compassion training programs could be operational levers for institutions aiming at fostering more compassionate HCPs–patients relationships.

## Introduction

The COVID-19 pandemic highlighted the suffering of Health Care Professionals (HCPs) at work, especially from work-related stress (Neto et al., 2020; Suryavanshi et al., 2020). Many elements of the medical culture and system contribute to permanent stress and pressure among HCPs, such as “individual blame, lack of organizational support, lack of community or family support, malignant training or work environments, perfectionism, personal coping styles […] encouragement of uncompromising standards, and promotion of prioritization of work and study over other areas of life” (Robertson and Long, 2018). The subsequent distress has been increasingly studied in the scientific literature over the last 20 years because of the resulting problems of burnout and its negative impact on patient care (Shanafelt et al., 2002; Thomas, 2004; Olson et al., 2021). This difficulty generally arises in the first year of training and increases during the course of the carrier and lingers (Dyrbye et al., 2006). Although burnout among HCPs can be caused by many factors (e.g., family stressors, personality factors), work stressors remain the most frequently cited causes and the ones usually considered as priorities to act on in the literature (Spickard et al., 2002). This is partly to maintain the health of HCPs, but also for patients as the quality of life at work of HCPs has an impact on the quality of care they provide and thus on the population’s health (Wu et al., 2011). The current health system contributes to a poor quality of life at work (i.e., many types of suffering and fatigue) of HCPs. Factors contributing to this dissatisfaction include long working hours and infrequent breaks, overwork, time pressure, limited control of work, difficulties in cooperating with other professions when caring for a patient, incompatibility of family and work obligations, insufficient income and low social support (Fuss et al., 2008; Witkoski and Dickson, 2010; Weigl et al., 2013; Domantay, 2014; Kunaviktikul et al., 2015; Zhang et al., 2018; Ortega-Galán et al., 2020; Ruiz-Fernández et al., 2020; Suryavanshi et al., 2020). This work-related unhappiness can have many deleterious effects on HCPs and patients such as negative emotional states (e.g., anxiety, depression), poor communication, work accidents and musculoskeletal disorders, compassion fatigue, sleep deprivation, poor nutrition, lack of exercise, and ultimately medical errors (Fuss et al., 2008; Witkoski and Dickson, 2010; Weigl et al., 2013; Domantay, 2014; Kunaviktikul et al., 2015; Zhang et al., 2018; Ortega-Galán et al., 2020; Ruiz-Fernández et al., 2020; Suryavanshi et al., 2020). In order to address the issues of diminished quality of life for HCPs and reduced quality of care they provide, mindfulness is increasingly cited as a therapeutic option whose constituent components (i.e., compassion, self-compassion, empathy) could address both of these concerns (Jermann et al., 2009; Singh et al., 2016a,b; Conversano et al., 2020; Nguyen et al., 2020; Zakeri et al., 2022). According to the United Kingdom Department of Health, *compassion* is a state wherein “care is provided in relationships based on empathy, respect and dignity; it can also be described as intelligent kindness, and is central to how people perceive their care” (Walker and Mann, 2016). Compassion can have benefits in many areas, and this is particularly the case for healthcare as it is considered a key to providing quality care to patients in need (Figley, 2002; Adams et al., 2006; Kret, 2011). However, HCPs are currently among the professionals considered most vulnerable to compassion fatigue and stress overload (Raab, 2014). *Compassion fatigue* can be defined as “the reduced ability or interest of the HCPs to empathize” or “bear the patient’s suffering” and corresponds to the “natural behaviors and emotions that result from knowledge of a traumatic event experienced or suffered by a person” (Raab, 2014). It has a negative influence on the quality of care. Stress, in turn, reduces HCPs’ ability to concentrate and focus (Miller et al., 1988).

*Self-compassion* is defined as involving “feelings of caring and kindness toward oneself in the face of personal suffering and involves the recognition that one’s suffering, failures and inadequacies are part of the human condition” (Birnie et al., 2010). Self-compassion includes three essential elements (Birnie et al., 2010): (1) showing kindness and understanding, rather than self-criticism and harsh judgment toward oneself; (2) considering one’s experiences as part of humanity as a whole rather than separating and isolating them; and (3) keeping painful thoughts and feelings in a balanced consciousness rather than over-identifying with them. Some researchers stated that the practice of meditation is a good way to enhance the qualities of self-compassion and empathy (Kristeller and Johnson, 2005; Newsome et al., 2012). Consequently, specific training programs for meditation and self-compassion have been developed. On the other hand, researchers are struggling to reach a consensus on the definition of *empathy*, which refers to the overall ability to take another person’s point of view and experience the resulting thoughts and feelings (Birnie et al., 2010). Thus, this skill includes both cognitive (accurately imagining another person’s point of view) and affective (emotional reactions to taking a point of view) components. Compassion is derived from the latter component, as it is expressed when one observes a person’s suffering and wishes to help him/her (Birnie et al., 2010). Notably, accurate empathy, as theorized by Rogers, is the most interesting empathy skill that must be developed by HCPs. Empathy includes a commitment to understanding the context surrounding a patient and the ability to communicate back to that patient what is understood about their request (Moyers and Miller, 2013). As such, it is considered to be a likely candidate as the “ultimate factor” for caring relationships (Moyers and Miller, 2013; Sturzu et al., 2019). It has been suggested that treatment outcomes would be improved if therapists are prepared to have authentic, empathetic relationships and possess an unconditional positive outlook toward their patients (Moyers and Miller, 2013). In fact, it explains more variance in outcomes than therapeutic alliance or type of intervention (Bohart and Greenberg, 1997; Moyers and Miller, 2013). Although empathy has a very positive impact on treatment outcomes, one must keep in mind that while empathy allows individuals to notice the pain of others, it also makes them more vulnerable to compassion fatigue (Raab, 2014). Therefore, this skill must be managed in association with other skills (e.g., being caring toward oneself) that help an individual limit such an effect. For these reasons, it is relevant to consider empathy as part of the basis of a good HCPs–patients relationship, alongside compassion and self-compassion.

We argue that HCPs’^1^ self-compassion and compassion toward others can be increased with the practice of mindfulness, specifically through healthcare-focused mindfulness training programs, such as the Mindfulness-Based Stress Reduction (MBSR) and the Mindful Self-Compassion (MSC) programs. *Mindfulness* is “a state of mind that permits insight, presence, and reflection, and also a habit of relating to the world” (Epstein, 2003a). It is often seen as a process that combines two components: (1) attention to both internal and external experiences in the present moment and (2) non-judgmental acceptance of emotions and thoughts (Sáiz-Manzanares and Montero-García, 2015; Conversano et al., 2020; Levit-Binnun et al., 2021). Practicing mindfulness (particularly as part of the MBSR program and the MSC) seems to be a good way of developing self-compassion among HCPs, as the changes in the ability to be self-compassionate are predicted by mindfulness-related changes (Miller et al., 1988; Neff, 2003). Notably, mindfulness develops prerequisite qualities for self-compassion and empathy (e.g., non-judgmental attitude, present-moment awareness; Neff, 2003; Beddoe and Murphy, 2004; Block-Lerner et al., 2007; Beckman et al., 2012; Verweij et al., 2018a; Malpass et al., 2019). Irving et al. (2014) showed that HCPs considered the practice of mindfulness to have opened their minds, allowing them to be more compassionate toward themselves and others (particularly toward their patients). The development of self-compassion is, therefore, essential to prevent compassion fatigue and promote compassionate care (Wiklund Gustin and Wagner, 2013). According to many researchers, including Figley (2002), the most effective HCPs are those who use and express compassion and empathy. Positive effects of mindfulness are also noticeable in interpersonal areas, as it develops empathic understanding and enables an unconditional positive relationship with the patient (Hick, 2008; Hoenders et al., 2016; Lases et al., 2016; Bentley et al., 2018; Verweij et al., 2018b). HCPs with higher levels of mindfulness tend to be more attentive and are more likely to use patient-centered communication. In turn, patients are more satisfied with this type of HCPs (Krasner et al., 2009; Beckman et al., 2012; Beach et al., 2013; Hoenders et al., 2016; Lases et al., 2016; Walker and Mann, 2016; Bentley et al., 2018; Verweij et al., 2018a). Attention and compassion toward the patient seem to be crucial for a good health professionals–patient relationship. Nonetheless, these elements can only lead to an optimal relationship with the patient if the HCPs is able to step back from their practice and become aware of their negative patterns (Miller et al., 1988; Sáiz-Manzanares and Montero-García, 2015). So, it is worth noting that mindfulness programs prepare trainees so that they can endure a large amount of suffering without being overwhelmed or becoming insensitive to patient suffering (Miller et al., 1988).

Mindfulness includes a set of methods that train the mind in order to reach a level of mental stability that fosters compassion and focused action (Epstein et al., 2008; Singh et al., 2020). This can help to fully awaken metacognition, a naturally present in everyone but hard to foster skill. It corresponds to an objective awareness of the internal events (mainly thoughts) and implies re-perception, which is the ability to change one’s perspective regarding current experience (Jankowski and Holas, 2014). Among the meta-cognitive experiences that can impact warmth and self-compassion is an attitude of benevolence and acceptance of current experiences, and seeing one’s experiences as part of the common human experience (Jankowski and Holas, 2014). Metacognition also allows HCPs to self-monitor themselves, i.e., to make it a practice to seek, integrate and respond to external and internal data on one’s own performance (Epstein et al., 2008). In this way, HCPs can assess their internal state and their anxiety about external actions and act on it (Epstein et al., 2008; Kabat-Zinn, 2016). Modern programs also help HCPs regain their calm in stressful situations. It can happen by helping HCPs become more aware of the stress they feel and to proactively set priorities and limits (Scheepers et al., 2020). It can also happen by reducing the tendency to strive for perfectionism and increasing their emotional intelligence. This, in turn, leads to greater compassion for patients (Dobkin and Hassed, 2016). Mindfulness can also help create a spaciousness in one’s experience that enables greater awareness and acceptance of “what is” (Raab, 2014). Also, the positive effects of these mindfulness trainings are long-lasting. In fact, Warnecke et al. (2017) proved that mindfulness training effects are long-lasting. Five years after mindfulness training, 88% of participants continued to use some form of mindfulness, meditation or relaxation exercise, of whom 63% specifically used mindfulness practices. Others, like Scheepers et al. (2020), claimed an effect of up to 6 years after implementation.

This practice thus seems as relevant for the well-being of patients as for that of HCPs (Ma et al., 2018; Jones et al., 2020). The burnout rate among doctors in France is between 38% and 52% (Shadili et al., 2018). *Burnout* is one of the most serious work-related problems in modern times and is a concept that is still being discussed in the scientific community. It can be defined as a process of emotional, physical and psychological exhaustion experienced by an individual when confronted with emotionally demanding work situations (Maslach and Jackson, 1981). We can identify three dimensions in this concept (Keidel, 2002; Angermeyer et al., 2006; Lazarescu et al., 2018; Zawieja and Benattar, 2019). *Emotional burnout* corresponds to the individual’s feeling of being physically and emotionally drained in the face of the emotional demands of their work. *Depersonalization* (or *dehumanization*) corresponds to the attitude of withdrawal and indifference adopted by an individual toward work and people in general. Finally, the *Reduction of personal fulfillment at work* corresponds to the individual’s feeling of professional failure and inability to meet the demands of the job. Burnout consequences include emotional (e.g., nervous tension, sad mood, etc.), physical (e.g., sleep disorders, chronic fatigue, etc.), cognitive (e.g., decreased concentration, difficulty performing several tasks at once, etc.), behavioral/interpersonal (e.g., withdrawal, social isolation, aggressive or even violent behavior, etc.), and motivational/attitudinal manifestations (e.g., disengagement, decreased motivation, etc.; Moyers and Miller, 2013). Thus, HCPs experiencing burnout become no longer able to have a meaningful relationship with their patients (Gueritault-Chalvin et al., 2000; Penson et al., 2000; Merlani et al., 2011; Lemoine et al., 2017; Lazarescu et al., 2018; Braun et al., 2019). Fortunately, a large body of literature has shed light on the positive effects of mindfulness on well-being and burnout prevention (Epstein, 1999; Epstein, 2003b; Hassed et al., 2009; Irving et al., 2009; Newsome et al., 2012; Conversano et al., 2020; Petitta et al., 2022), indicating that it leads to a feeling of being able to perceive the return of dysfunctional coping patterns and take a step back from negative events. Thus, empathy, compassion and metacognition seem to be at the core of an optimized mindfulness practice. However, these three components are rarely found in the same program (however, while not directly offered with meditation in the programs, empathy is transmitted through instructor-trainee relationship). To our knowledge, no programs have been developed to better understand the impact of vocational training in empathy, compassion and metacognition through mindfulness on the HCPs-patients relationship. Thus, we decided to design a program dedicated to this purpose and to investigate its effects on healthcare reported by HCPs. We hypothesized that a program of this kind could improve patient–health professionals relationships and the care provided by the latter.

## Methods

### Description of the program

**Figure 1:**
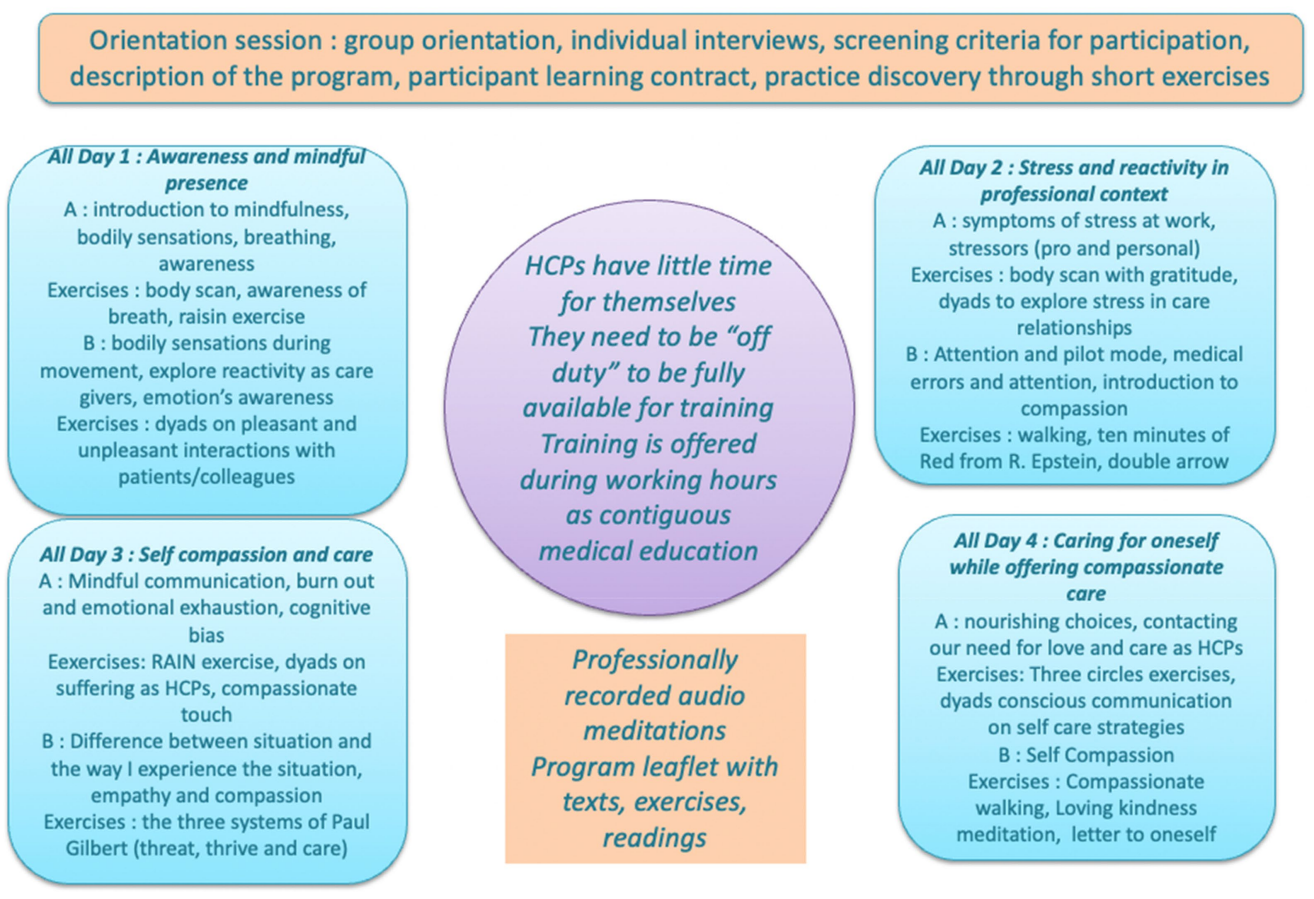
MB CARE’ curriculum.

The MB CARE program, drawn from the MBSR and MSC training programs, is a curriculum dedicated to HCPs aiming at reducing burnout level, while enhancing patient centricity by training HCPs critical qualities of mindfulness and self-compassion (Isnard, 2019). In this sense, MB CARE differs from the MSBR and the MSC as it is an integration of these approaches adapted to the practice of HCPs. The program integrates three themes, namely, mindfulness, compassion and self-compassion. Mindfulness training focuses on the development of a non-judgmental attitude and good attentional skills (Eddy et al., 2015). The compassionate component is to enable HCPs to embrace their imperfection and accept themselves as human beings. Delivered by experienced MBSR/compassion instructors (not involved in the research), the MB CARE program offers meditation and compassion exercises, dyad communication training, and guided dialog with the same pedagogical frame that MBSR (Figure 1; Table 1). An essential feature of this program is the exercises’ focus on professional daily life stressors, interpersonal communication and stressful emotions that HCPs actually experience in their practice, in a uniquely recontextualized meditation experience. The program follows an incremental overview of practices. First, learners work on personal themes before practicing with patient or work-related themes. It features exercises on the body and sensations, followed by discussions about pleasant/unpleasant experiences combined with thoughts and bodily sensations, while putting trainees back into situations where they have experienced these sensations on their own. The issue of stress is introduced through work on situations where HCPs experience difficulties with others. As a new feature among the mindfulness training programs being implemented, the MB CARE program directly adapts the practice of mindfulness and compassion to professional situations that are relevant for HCPs, either focusing on HCPs-patients relationships or in between professionals. As in the MBSR program, the agenda and objectives were not communicated to the trainees so that they experience living in the moment through experiential pedagogy. Also, MB CARE did not offer a 1 day long intensive meditation practice day (i.e., as in the MBSR program). More details about the content of the program can be obtained by contacting the authors.

**Table 1:** Themes covered by the MB CARE program.

Themes	Content
Cognitive	Attention/awareness, disengagement, critical thinking, non-judgmental thinking
Physical	Body awareness/breath awareness, reconnecting appropriated use of body signals to experience emotions while in empathy with patients
Emotional	Emotions awareness in relation to therapeutic relationships, reducing reactivity, empathy/compassion/emotional exhaustion
Behavioral	Impulse control/deconditioning
Relationship to self and others	Connectedness acceptance/improving self-acceptance and forgiveness, self-compassion/loving kindness practices, caring practices, game roles
Spiritual intelligence	Inner peace/increasing sense of connection with wiser thinking

The MB CARE program was introduced *via* an announcement in the internal service of the *Faculté de Medecine Pitié Salpetrière that* informed HCPs that a new program promoting a health professionals–patient relationship based on compassion and empathy would be implemented (i.e., the MB CARE program). The HCPs were invited to register on a voluntary basis through a large communication conducted *via* institutional newsletter (35,000 email addresses) or through a continuous medical education program in the community. Because HCPs had to be fully engaged into the program and be perfectly available, we chose to offer the program in full day sessions and not weekly 2 to 3 h sessions. The HCP’s were totally off duty on those days. Once participants agreed to take part in the program, they received an email informing them that a study-which would take the form of a one-on-one interview—would be conducted to evaluate the impact of the program on their professional practice. They were invited to accept or refuse to participate in this study by return email. Participants followed the program according to the following modalities:

- Program implementation of 4 days in a row (*n* = 4) vs. once a week for 4 weeks (*n* = 7);
- At the hospital (*n* = 7) vs. at the Espace City’zen (*n* = 4; i.e., a place dedicated to the practice of yoga, meditation and personal development located at the Parc Floral in Paris);
- Paid (*n* = 4) vs. free training (*n* = 7);
- Experienced (*n* = 5) vs. inexperienced (*n* = 6) in mindfulness (i.e., if the person has ever practiced mindfulness before the training); and
- Training given by C. (*n* = 4) vs. Training given by T. (*n* = 7; i.e., the two trainers teaching the program together with CB).

### Our study

#### Inclusion and exclusion criteria

We conducted our study after the delivery of the MB CARE sessions. For participant recruitment, we used a relevant convenience sample of former participants who were contacted through their email addresses that they had provided to the MB CARE organizers during their participation. Participants were 11 HCPs who had fully completed the program (M_age_ = 43.55, ET_age_ = 9.50, 10 women, 1 man) at least 1 year before the interview period to assess the long-term effects of the program. They were all experienced HCPs (over 10 years of practice) as defined in the Public Health Code^2^ that participated on a voluntary basis. Five of them had already practiced mindfulness, five had not and one did not specify. There were three nurses, six doctors, one social worker and one nurse manager.

#### Design of the study

We conducted an exploratory qualitative study using semi-structured interviews to assess the impact of the MB CARE program. All interviews were conducted between May and July 2020. Due to the sanitary context during the study, all interviews were conducted using phone calls. The interviews were conducted by one of this article’s authors and were recorded and later fully transcribed for the accuracy of the material gathered. At the interview’s beginning, the participants were informed that the purpose of the interview was to assess the program’s impact on work practices and, more specifically, the health care professionals–patient relationship. It was clarified that the interview was unrelated to current events and the global health context of COVID-19. For each person, we used the same interview grid that was especially designed for the study. The interview grid (Appendix 1) was built around three main themes:

1. The experience of the training (i.e., satisfaction and potential improvements);
2. How HCPs take care of themselves (i.e., what are their stressors and support systems); and
3. How the program has impacted the practice of HCPs (i.e., in their relationship with patients). This third category had three sub-categories:
  - Impact on practice (i.e., better work balance and mindfulness practice),
  - Impact on metacognitive skills (i.e., emotion regulation and reflective thinking), and
  - Impact on the participants’ empathy skills and the therapeutic relationship (i.e., empathy and compassion toward oneself and toward the patient).

The grid had two purposes. First, it provided answers to the questions raised in the introduction. Second, it allowed for the assessment of the four levels of Kirkpatrick (1959) training evaluation model. This is the reference model in this area, particularly because it is broad enough to correspond to the needs of professionals for all types of training (Santos and Stuart, 2003; Bates, 2004). The first level, “Reactions,” assesses learners’ satisfaction with the training (What did the learner like about it?). We asked questions such as “What did you like (What were the criteria for satisfaction: content, teaching methods, etc.)”? The second level, “Learning,” evaluates the knowledge that the learners will keep (What will they have learned at the end of the training? What knowledge, know-how and interpersonal skills will they have gained?). HCPs were asked: “What did you learn during the training”? The third level, “Behaviors” (or transfer), is an assessment of the behaviors that the learners will be able to transfer from training to their work practice (What new behaviors have they learned? Do they use them in their workplace?). We asked: “How do you take care of yourself”? Finally, the fourth level, “Results,” evaluates the results at the end of the training in terms of economic gain, profitability, productivity, and so on. We did not ask questions at this level, as profitability was not a goal of the program. It should also be noted that we did not assess participant’s personal involvement in practicing mindfulness or compassion.

The interview always ended in the same way. We asked the participants how they felt. We also reminded them of the purpose of the interview and asked for any additional information that they felt would help meet that purpose and had not been addressed during the interview.

#### Ethical aspects

This study received a favorable ethical decision from the INSEAD-Sorbonne University Behavioral Lab, ensuring, in particular, compliance with (1) of the Declaration of Helsinki criteria and (2) the French law regarding personal data protection in research studies in health sciences. Before the interviews, the participants’ willingness to be involved in the interviews was provided through email written consent. During the interviews, consent was ensured by asking if participants agreed to take part in the interview and have their data processed afterwards, and they were reminded that they were free to accept or refuse to answer each question.

### Results analysis procedure

When transcribing the recorded interviews into Word documents, each interview was anonymized by being assigned a number (e.g., Interview 1). We chose to remove one person’s interview from the analyses because of irrelevance. We made sure that the transcriptions accurately reflected what the participants had said. We analyzed these interviews using King’s template analysis method (King and Brooks, 2017). Each question within each topic was analyzed, and we selected the most relevant verbatim statements for each question. These were considered relevant when they were representative of an opinion expressed by the majority or when they raised important issues for future program development. Specifically, we inputted the interview grid into an Excel spreadsheet. For each question, we created an “Answer” column. For every interview, we took the section where a question was addressed and chose one or more verbatim that expressed in the most representative way what the participant thought about the question asked. Verbatim was then included in the “Answer” column of the respective question. Like most similar research, the data was analyzed by a single researcher. The credibility of results was then assessed by ensuring that verbatims were representative of the interview findings (Elo et al., 2014). We went back and forth between the questions and the interviews to make sure we were capturing what the participants reported. This method seemed relevant to avoid common problems such as over-reliance on qualitative software packages, word overload due to line-by-line approaches or difficulty of retaining the integrity of each respondent’s story (Dierckx de Casterlé et al., 2012).

## Results

### The training experience

Overall, the training was considered to be a very enjoyable experience: “The experience of the program [was] very pleasant” (Interviews 5 and 9). Only one person found the experience in itself difficult, pointing out the resulting effects as positive. When participants described what they liked about the training, they brought up the small group size: “What I liked a lot was that we were a very small group” (Interviews 2, 5, 7, 8, and 9). This encouraged sharing and allowed participants to realize that other HCPs shared their difficulties, without feeling ashamed or embarrassed, because everyone understood the situations they were facing. The majority of the participants perceived the program not as professional training, but as personal development training that could have indirect positive effects on their practice afterwards: “It is personal development at the service of professional development” (Interviews 10 and 6). Only one person, who had been meditating for many years, considered this training to be fundamentally professional: “Obviously it involves the whole being, both in its private and professional dimensions, but the internship is very structured on the care relationship, it is very structured on work, it is really centered on that. So it is 100% professional training” (Interview 5).

When asked what they would like to change in the program, the participants often mentioned a need for post-training follow-up and reminders: “So what I would like is to have something after […] a reminder” (Interviews 5, 6, 7, and 10). One person also reported that the program should offer more intersessional follow-up to encourage the sharing of feelings and changes that they were experiencing. Finally, some people mentioned the duration of the training, which was considered too short to have a long-term impact: “Afterwards, I sincerely think that 4 days of MB CARE training will not be enough to develop caring listening skills. If the people who follow this training do not continue on this path, well, I am not sure of the impact on their daily professional life” (e.g., Interview 9).

### The impact of the MB CARE program on self-care

The participants mentioned a variety of stressors that hindered their well-being in their daily work, such as their work environment and hierarchy [“Having several demands at the same time” (Interview 2); “It is organizational stress” (Interview 5)], and psychological factors related to self-confidence and overwork [“The fear of not being competent and that the signs of my lack of confidence are legitimate” (Interview 6); “It is the workload, the diversity of things I have to do” (Interviews 3 and 10)]. A large part of the participants reported that their self-confidence had evolved following the MB CARE program, and that they allowed themselves more room for error: “It allowed me to evolve, to have more confidence in myself” (Interviews 4, 3, and 2); “I’m now less a perfectionist” (Interview 4). Nevertheless, some noted that this confidence was still fragile: “My confidence in myself is fragile and needs to be supported and encouraged” (Interviews 1 and 6). Participants also reported practicing multiple stress-reducing activities, for example, “By taking breaks […] By trying to give myself time […] I take care of myself with meditations at home, which are more formal practices and then sports” (Interview 5). They also mentioned the exercises they learned in the program, such as the use of breathing techniques.

### The impact of the MB CARE program at work

We evaluated the impacts of the MB CARE program along three axes: the impacts on HCPs’ professional practice, on their metacognition skills and on their empathy/compassion/self-compassion skills.

Interestingly, one participant noted the importance of the HCP’s emotions in providing optimal care: “I realized how much my positioning or emotional state could influence the quality of the relationship” (Interview 5). The program appeared to have taught the participants to remain calm in stressful work situations, including those who did not continue practicing meditation. The participants also mentioned a greater capacity to take a step back (i.e., to put distance between themselves and the occurring situation). According to them, the training helped them to manage strong emotional feelings and to reflect on their emotions, thereby enabling them to react in a more appropriate way: “So the MB CARE training has taught me a lot, like to take a step back, to try to understand what state I’m in, what’s going on inside me, that’s how I can better face things” (Interview 10). The program seems to have enhanced participants’ ability to balance their emotions and reason as well: “Trying to respond to the situation rather than reacting according to one’s emotions” (Interview 3). They were also better able to realize when they were immersed in their emotions and thoughts: “So it happens to me sometimes in extreme situations, but it does not last long. I notice it pretty quickly” (Interview 4). However, a number of participants were still overwhelmed by their emotions, sometimes at the expense of their being grounded in reality. They mostly used breathing methods to manage their emotions and defer their effects to give themselves time to reflect. However, this kind of practice seemed to only be effective under certain conditions, such as anticipating the situation: “If I can anticipate the situation, the problem, yes. But if I really get caught up in a situation where I know I’m going to escalate, no.” (Interview 2). The participants also reported being more able to manage their workloads in order to make time for themselves: “It did not necessarily change my relationship with work, [but] it changed the way I perceive the effect work has on me” (Interview 5). This tendency to take better account of one’s limits and needs was noted when participants talked about burnout. The program seemed to have helped them to become aware of “risky” situations as well as to accept their human condition and an ability to let go: “I accept that when I’m tired I’m less efficient and that, therefore, it is necessary to stop and hand over work” (Interviews 6, 9, and 10). Moreover, many of the interviewees spoke of increased caring listening skill: “I am more attentive to what the other person says to me, I leave room for the other person, I really listen to them” (Interview 2). The participants paid attention to their patients, especially through the identification of perceptual clues. The former also mentioned paying more attention to the latter by managing their priorities: “Well, I’m getting better […] I can see what is a priority and what is not” (Interview 4). However, there were those who said that they did not really pay more attention to themselves or the patient in their interaction. The MB CARE program also reduced the participants’ tendency to be judgmental: “My view of others, of life, of myself, of judgment, all of this has changed” (Interview 4). The interviewees explored their feelings and emotions to a greater extent, and the majority were more accepting of them. This included acknowledging the emotion and accepting its existence: “That was quite new during the training. To tell myself I can recognise emotion without judging it and without asking myself why it is there […] I do not inhibit it, I regulate it” (Interview 10). The participants were more likely to regulate them than inhibit them. A few of them, however, felt that they were still judgmental toward their emotions, which they considered to be inadequate and not required. These people were the ones who preferred to inhibit those emotions: “I cannot welcome them without judgment. I will consider myself as weak, as fragile if I still feel fear or anxiety” (Interviews 1 and 3). The program also helped the participants to live more in the present moment: “There you are, you are really in the moment, you are listening to your body, you are in what’s happening right now for you, and it helped me to put things into perspective and get back to work” (Interview 4). They reported refocusing through short meditative practices (e.g., focusing on sensations): “I realize that I am doing some sort of micro meditation. It means micro refocusing on the present moment” (Interview 6). Furthermore, the program appeared to have had a great influence on participants’ ability to concentrate: “It allowed me to better identify my needs and, therefore, better focus on what I’m doing” (Interview 2). More specifically, the program impacted participants’ ability to focus on one task at a time: “I stopped doing two things at the same time […] That’s one of the big points of the program. To understand that actually I’m like everyone else—I’m not able to do two things at the same time” (Interview 8). Many participants talked about exploring their sensations as a way to refocus on the present moment, including vision, hearing and taste: “I’m reconnecting to the heat […], a breeze, some air, a ray of sunlight […] I’m going to be more attentive to the texture, the taste, of what I’m eating” (Interview 1). These sensations were also used during the professional practice to monitor one’s state: “When there is something that I am experiencing that is too intense […] I feel it quite quickly and I sit down. And I take a deep breath” (Interview 4). Nevertheless, two participants reported not being able to explore their sensations, especially during difficult experiences at work. When participants talked about their current practice of mindfulness, this mostly included informal practices, such as micro-meditations in the workplace: “I practice in informal micro meditation all day long, but not ritualized” (Interview 6); “On stressful consultations, I try to take 3 min afterwards to try to refocus on myself” (Interview 9). This informal practice was often coupled with formal practices, especially between consultations. The participants mostly practiced meditation for short periods of time, ranging from 10 to 30 min, often daily: “I try to do 10 to 15 min of breathing techniques. It’s going to be at home [where I] do a scan, do a compassionate meditation” (Interview 9). The body scan was often cited, but some found it rather unpleasant. Regarding the equipment used, two people mentioned a guided meditation application: “I practice it with the Petit Bambou application” (Interviews 1 and 3). However, two participants had abandoned this practice. Others said they often thought about practicing, especially when they experienced difficulties, but did not do so: “When I feel that I am beginning to be overwhelmed, when I feel that I am starting to feel emotions that are too intense, that tend to parasitise me, then I think about it” (Interview 6, Interview 1).

The majority of the trainees reported that the program improved their ability to understand patients’ perspectives in order to better accept situations: “Well, if I had someone talking to me like that in front of me, what would I understand? […] Do I get the impression that he is interested in me or that he is not interested in me.” I find this change of perspective interesting […] It allows me to see my position in relation to the patient’ (Interview 5). Some participants mentioned conditions that affected their ability to change their perspective, such as the emotional intensity felt at the time. Some participants showed signs of developing their metacognitive capacity, but were not able to express it clearly: “[There is] a lot of confusion at first […] I try to think about situations at the same time and then I try to reassure the person […] There are a lot of things going on in our heads, I cannot necessarily identify them, I recognise them” (Interview 10). One person mentioned the possibility of analyzing one’s thoughts afterwards in order to react in a more appropriate way in the future. Regarding decision-making, the majority of the HCPs considered that they were aware of the decisions they made: “Well, when I make a decision, I usually know” (Interview 4). For some, they considered that they now took more time to think about it: “It has evolved a bit, because at the beginning I tended to make decisions from scratch […] I’m going to give myself time to think about what I’ve been asked to do, or what could be done” (Interview 1). However, their decision-making remained very factual and did not yet demonstrate high-level metacognitive abilities. One participant even said he did not think anything through during the decision-making process. Much of the thinking seemed to come after the decision was made: “Well, there is always a questioning, ‘Did I make the right decision?’ and a questioning of ‘I did not make that decision’” (Interview 7).

The HCPs felt that they were kind to their patients: “I think I am kind to them” (Interviews 1 and 7). They also felt that the program had helped them to be kinder to themselves, which they thought had positive repercussions for their patients: “With MB CARE’s meditation approaches, I try to be kinder to myself and it helps me to be kinder to others as well” (Interview 3). Some, however, stated that they were already kind to their patients even before the training. A few expressed the fact that the term “kindness” was not appropriate, pointing out instead the characteristics of a warm relationship: “I do not think “kindness” is really the right term […] maybe a lot of things that might pass for kindness from the outside, but I think it’s not really the same” (Interviews 6 and 10). Overall, the participants were still self-critical but considered this criticism to be constructive and benevolent: “I am still self-critical but in something constructive” (Interview 10). This self-criticism also seemed to encourage acceptance of boundaries: “What MB CARE has done for me is that before, when there was a conflict, I always told myself that I was the only one responsible […] And now I tell myself, “Well, maybe.” Anyway, there is always responsibility on your part when something goes wrong, but maybe there was no solution anyway” (Interview 6). Only one participant stated that they never criticized themselves anymore and were strongly opposed to it. The interviewees were generally more benevolent toward themselves. The majority of the respondents considered that they were able to feel the pain of their patients: “I share their suffering a little and without feeling it […] Sometimes, it can shock me […] there is a form of acceptance” (Interviews 4 and 7). Some indicated that the program did not enable them to feel the patients’ suffering, but that it had helped them identify and perceive it: “I imagine how I would feel if I felt the same suffering, but it is not the reality of his suffering, because I am convinced that the reality of his suffering is something only he can feel” (Interview 5). The participants also seemed to be more accepting of their patients’ negative experiences. Some people pointed out a greater ability to distance themselves from patients when necessary: “So it may seem a little negative, but I am not always available for them” (Interview 1). However, two participants considered that there had been no changes in their relationships with patients. Finally, reflecting an empathetic image was important to the participants. It seemed to be the cornerstone of the HCPs-patient relationship for them: “I hope they have a warm and empathetic image of me. It’s part of the job” (Interviews 3 and 4). Interestingly, one participant mentioned the use of empathy and compassion as a practical resource to be consciously integrated into the HCPs-patient relationship: “If we manage to put empathy and compassion in even a 2/3 min regulation, it changes things completely, but really fundamentally” (Interview 5).

## Discussion

The growing concern regarding stress and burnout in the workplace raise the question of the quality of life of HCPs and the quality of the care they provide. In view of this situation of HCPs’ work-related stress and the resulting negative consequences on patients’ health, many authors have called for the development and generalization of emotional resource management programs to cultivate, among other things, the compassion of HCPs and prevent compassion fatigue and burnout (Ruiz-Fernández et al., 2021). Indeed, compassion is considered a key factor in providing quality care to patients in need (Miller et al., 1988; Kristeller and Johnson, 2005; Birnie et al., 2010). However, HCPs are currently among the professionals most vulnerable to compassion fatigue and stress overload (Raab, 2014). We know that encouraging HCPs to develop a patient-centered care relationship has beneficial effects for both the patients and their HCPs (e.g., decrease in symptoms of depression, depersonalization, and emotional burnout, and increase in emotion regulation skills, higher levels of self-care, and enhanced communication skills at work; Isnard, 2019). The literature suggests that HCPs’ compassion and self-compassion can be increased with the practice of mindfulness, more particularly through caregiving-focused mindfulness training programs (Neff, 2003; Birnie et al., 2010; Bentley et al., 2018). Those programs are particularly relevant for HCPs, who have a greater risk for professional burnout due to the high level of involvement required. Thus, we hypothesized that a mindfulness training program specifically focused on situations that are relevant for HCPs could improve patient-health professionals relationships, and ultimately, the care provided. We conducted a qualitative study designed to explore the perceived psychological consequences of such training programs among HCPs who experienced mindfulness and compassion training (i.e., the MB CARE program). We conducted interviews with 10 HCPs who had fully completed the MB CARE program. Our objective was to evaluate the impact of this type of practice-based training through the study of the capacity of HCPs to apply its methods in their daily practice.

**Figure 2:**
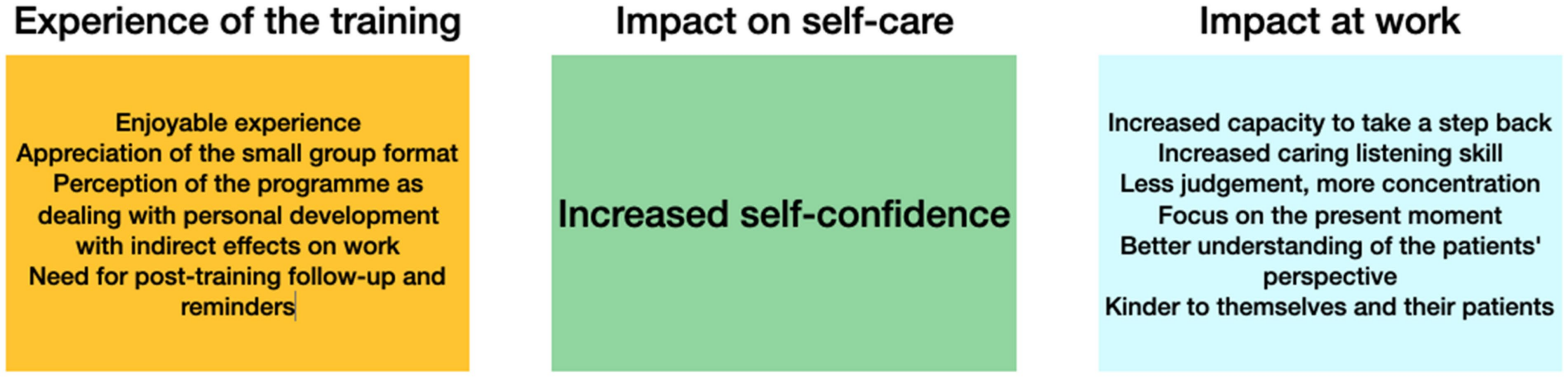
Impact of the MB Care program by domain of the interview grid.

Our content analysis results showed that the training seemed to have an overall positive impact on the HCPs’ ability to take care of themselves, particularly through the ability to “take a step back.” It appears to have helped the participants to remain calm in stressful situations. In terms of their relationship with the patient, caring listening seemed to be one of the main skills that the program helped the HCPs to develop. Participants seemed to use mainly informal practices in their workplace. In comparison, formal practices were preferred at home, in time slots not exceeding 30 min. The program also appears to have enhanced the participants’ ability to recognize the presence of their emotions, which were better accepted—especially negative ones—with benevolence. The participants also seemed to be able to change their perspective in order to better understand their patients. They were more attentive to their patients and their needs, without being able to articulate how well they were paying attention to them. The participants felt they shared their human nature with their patients, although they still acknowledged their individuality. The majority considered themselves empathetic and thought they could perceive their patients’ suffering without feeling it. The program also facilitated the development of kindness toward themselves and their patients. Overall, the participants were better able to accept the difficult experiences they encountered in the workplace or those reported to them by their patients. A graphical summary of the results can be found in Figure 2. Thus, we conclude that professional mindfulness training programs could be operational levers for institutions aiming at fostering a more compassionate HCPs–patients relationship, ultimately ensuring the efficient provision of care and well–being of both HCPs and patients. However, it is worth noting that those claims are relying solely on the perceptions of HCPs and do not engage the feelings of their patients.

### MB CARE’s main point

One of the most crucial elements in this series of interviews was to determine whether HCPs considered the MB CARE to be a vocational training or a personal development program. The respondents offered a variety of responses, ranging from viewing it as a full personal development program or as a very grounded, practice-oriented training. However, the modal response is that it is a personal development program that can have positive consequences for the HCPs–patients relationship. The MB CARE program, therefore, seems to be perceived as a kind of support that enables HCPs to better cope with their practice and protect themselves from problems related to personal exhaustion and lack of consideration for their limits. Exerting efforts to better understand their needs and listening to their feelings would enable them to be more empathetic toward themselves and, indirectly, toward others, thus resulting in compassionate care. These results are very encouraging insofar as HCPs are able to see the value of the program for their professional practice and for the majority of them to use those teachings for the benefit of their HCPs–patients relationships. It now remains to be established whether there is a need for more emphasis on the vocational aspect or whether this perception of the program as personal development with indirect vocational effects is sufficient to have truly positive effects on their relationships with their patients. Future studies of other practice-based and compassion focused programs should be undertaken to determine what adaptations are needed.

### MB CARE’s future improvements

One potential area for improvement would be a focus on the development of metacognitive skills, as many participants expressed difficulties in this area. The development of HCPs’ metacognitive abilities was notably intended to enable them to better identify the internal processes involved in their practice and to foster their capacity for empathy. However, many HCPs were not able to demonstrate significant metacognitive abilities. The majority of the respondents were not able to spontaneously express any internal processes, or when they did, it was very superficial. This could bring us back to the vision of meditation as an ongoing training that needs to be nourished by regular practice, a classical condition for self-development through mindfulness. These results could also suggest that the MB CARE program, based on MBSR may inherently lack impact on metacognition. Hussain (2015) highlighted the independent development of mindfulness and meta-cognition theoretical frameworks, and suggested that some specific mindfulness-based interventions such as detached mindfulness (Hussain, 2015) were more likely than others to enhance metacognitive abilities. Similarly, Corti and Gelati (2020) were able to enhance metacognitive abilities with Mindfulness Effective Learning. This suggests that extending the scope of MBSR-based-programs to other mindfulness-based protocols could be useful to specifically improve participants’ metacognitive abilities.

### Conclusion

The development of more compassionate health professionals–patient relationships through mindfulness is still in its infancy. This study is an encouraging first step toward more comprehensive programs that are, hopefully, attended by more HCPs. Our goal is to adjust the program so that it better aligns with the areas for improvement that we have identified. Thus, we plan to conduct further series of evaluations in order to validate the positive effects of the MB CARE program for developing a caring relationship based on compassion and empathy while preventing burnout. Moreover, mindfulness impacts care through other constructs. As we have seen here, metacognition is particularly of concern, but this could be the case for other concepts as well, such as for example critical thinking. Since the work of Hofling et al. (1966), it has been known that HCPs can sometimes listen to the instructions they are given without being critical. Many researchers currently believe that the development of critical thinking skills is an important factor in improving the quality of care. This is notably the case of Facione et al. (2017), who stated that: “Nurses with strong critical thinking do not function as clinical robots.” As good critical thinking requires excellent metacognition skills, it seems that an interesting avenue is to develop these linked constructs in order to promote quality care. This is but one of the many constructs impacting care on which mindfulness can have a positive effect. To conclude, our results demonstrate that mindfulness and compassion could become relevant tools for fostering healthy, empathetic and compassionate HCPs–patients relationships, contributing to the well-being of both HCPs and patients. We hope that more research will be done to identify the levers that will allow mindfulness and compassion programs to become a key component of professional training for HCPs.

However, some limitations should be noted in this study. The main limitation is due to the time lag between the program and the investigative phase of the interviews we conducted. The program was conducted in 2018 and 2019, and we carried out the interviews in 2020. Therefore, for the participants, 1 to 2 years had elapsed since they had completed the program. This implies that their memories were very fragmented, and only the most vivid memories remained in their minds. In fact, many participants brought this up spontaneously, as noted: “This program was [implemented] a long time ago.” However, one can argue that this period of time allowed professionals to implement real changes in their practice following the program. Furthermore, it is important to note that participation in the MB CARE program was voluntary. Therefore, the interviewees had a certain degree of interest in a humanistic perspective in their work. They were also those who suffered the most in their daily practice. Individuals who do not feel this kind of distress generally do not turn to this type of training. Our study, therefore, focused on HCPs who may not be wholly representative of the HCPs population. We cannot guarantee that comparable results would be observed in individuals who had no particular interest in the mindfulness approach. Thus, future studies could address this issue and consider those related to adherence to programs without prior motivation and issues of engagement and reactance. Because participation was voluntary and few participants had the opportunity to attend the MB CARE program, the study was conducted on a relatively small sample of HCPs. We believe that this sample is of sufficient size to discern patterns of impact of MB CARE, but future studies should include larger samples, possibly of 20 or even 30 HCPs. Indeed, we consider to have reached good data saturation on results referring to the HCP-patient relationship (e.g., impact of mindfulness practice on the ability to take a step back). However, a larger sample would make our findings more robust, or be more likely to highlight between group (e.g., specialty, managerial position, etc.) differences. In addition, we did not make any selection in regard to the HCPs’ professions, which gives access to a sample of HCPs in different positions with similar problems but with significant disparities (e.g., pediatricians have to handle the relationship with the parents of their patients more than that with their patients). Thus, future studies should propose methodologies that ensure better control of samples, allowing conclusions that can be better generalized to specific HCPs populations. Furthermore, we did not control for the level of pre-program mindfulness practice among the participants. This was noticeable in the interviews, as some participants emphasized the richness of this first experience, without really being able to describe how the training had changed their practice, while others were unable to tell us about the specifics of the program, because they already had so much experience in this area. Unfortunately, we do not have precise data on mindfulness training participants may have received before, during, or after MB CARE (i.e., exercise type, practice duration). Future studies should collect this information in order to accurately determine the effects of a program comparable to MB CARE in conjunction with other mindfulness programs that participants may have taken. Besides, we decided to look at the overall impact of MB CARE without distinguishing between the different modalities available in the program (e.g., we did not distinguish our analysis by the type of person who delivered the program). This is because we wanted to assess the impact of MB CARE as a whole. However, the training experience section of the interview grid was intended to help identify whether there were any differences in the program that were important. Further studies could investigate, among other things, the variation in results according to the location of the training, the type of trainers, or the timing of the training. Also, the very focus of the program means that we have little insight. Indeed, as previously stated, many mindfulness programs have demonstrated positive effects. However, as programs that specifically develop compassion are, to our knowledge, currently very under-represented, it is difficult to make comparisons. Therefore, the results of this study cannot be compared to other similar studies. Furthermore, if participants were recruited with achievement of data source triangulation in mind (Patton, 1999), all of the perspectives considered (i.e., nurses, doctors, social worker, nurse manager) were on the HCPs’ side. Future studies will need to compare their perceptions with those of their patients to see if there is any agreement.

## Data availability statement

The raw data supporting the conclusions of this article will be made available by the authors, without undue reservation.

## Ethics statement

The studies involving human participants were reviewed and approved by INSEAD-Sorbonne University Behavioural Lab. The patients/participants provided their written informed consent to participate in this study.

## Author contributions

CB: conceptualization, methodology, investigation, formal analysis, and writing-original draft preparation. AA: supervision, conceptualization, methodology, formal analysis, and writing-reviewing and editing. LH: conceptualization, methodology, formal analysis, and writing-reviewing and editing. CM: investigation. TC: investigation. CI: supervision, conceptualization, funding acquisition, and writing-reviewing and editing. All authors contributed to the article and approved the submitted version.

## Conflict of interest

The authors declare that the research was conducted in the absence of any commercial or financial relationships that could be construed as a potential conflict of interest.

## Publisher’s note

All claims expressed in this article are solely those of the authors and do not necessarily represent those of their affiliated organizations, or those of the publisher, the editors and the reviewers. Any product that may be evaluated in this article, or claim that may be made by its manufacturer, is not guaranteed or endorsed by the publisher.

## Footnotes

^1^As defined here: https://www.vie-publique.fr/fiches/37855-categories-de-professionnels-de-sante-code-se-la-sante-publique

^2^As defined here: https://www.vie-publique.fr/fiches/37855-categories-de-professionnels-de-sante-code-se-la-sante-publique
