# Supplementary material for: Mindfulness and compassion training for health professionals: A qualitative study
Source: Front Psychol. 2023 Jan 12;13:1113453. doi: 10.3389/fpsyg.2022.1113453 (PMC9878613; doi:10.3389/fpsyg.2022.1113453)
Supplement: Supplementary file 1 [file Presentation_1.pdf]

Appendix

Appendix 1 : Interview Grid

Introduction to the interview

Hello and thank you for coming. I am ---- and I am conducting interviews on the MB CARE program in order to evaluate its impact on professional practices and more specifically on the caregiver-patient relationship.

I am going to ask you various questions on this subject: please note that everything that is said during this interview will remain anonymous and confidential. You are of course entirely free to accept or refuse to answer each of my questions. You should also know that this interview is not related to current events and the global health context.

Do you agree that I may record our exchanges in order to facilitate the transcription? I will be the only person to have the recording and will delete it as soon as the transcript is complete.

Checking the independent variables invoked

Among the modalities I am going to mention, which ones are related to the training you attended?

- Place of training: Zen space vs. hospital,
- Organization of days: 4 days in a row vs. 4 independent days separated by a week,
- Public: Initiated (DU etc) vs. non-initiated,
- Finances: Paid vs. free,
- Presentation: Training vs. clinical study,
- Trainers: Corinne + Claire or Thierry vs. Corinne,
- Context: All contexts vs. Public assistance.

Themes and follow-up questions

Initial open question:

How did you experience the MB CARE program and how has it impacted your professional practice?

Theme 1: Training

Open question to address the theme:

Can you tell me about your experience of this training program?

Follow-up questions to explore the theme:

- How did you come to want to participate in the training?
- How would you define this experience if you were to tell someone who had never heard of this training?
- How did you experience the training?
- What did you like (what were the criteria for satisfaction: content, teaching methods, etc.)?
- How did you experience learning mindfulness in a small group?
- What did you learn during the training?
- For you, can this training be considered as professional training or more as personal development?
- If you had to change something in this training, what would you do and how? (regarding the content, the format, the deployment).

Theme 2: Caring for yourself as a caregiver.

Open question to address the theme:

How has this training impacted your relationship with yourself and the way you care for yourself?

Follow-up questions to explore the theme:

- What are the stressors in your daily practice?
- How do you take care of yourself?
- How do you deal with adversity?
- How do you deal with strong emotions
- How do you deal with self-confidence
- How do you deal with the separation of life domains
- Do you seek support when you need it? In what way?
- Have you ever asked your colleagues for help and encountered obstacles? What obstacles?
- How is it to seek help in the workplace?
- Do your colleagues express particular emotions?
- Do you feel support from peers?

Theme 3: MB CARE in current care practice

Sub-theme 1: Practice

Open-ended question to address the sub-theme:

Does what you have learned during the MB CARE training program have a place in your daily work and if so, what is it?

Follow-up questions to explore the sub-theme:

- How has this training changed your practice?

  If so,

- How does what you have learned in the MB CARE program enable you to decrease your burnout?
- How has what you have learned in the MB CARE program changed your relationship with work?
- How has what you have learned in the MB CARE program changed your relationship with patients?
- How has what you have learned in the MB CARE program changed your interactions with your patients and the way you approach care?
- How has the MB CARE program helped you to gain the ability to balance reason and emotion?
- How has the MB CARE program helped you to gain the ability to adopt a non-judgemental stance?
- How has the MB CARE program enabled you to gain the ability to create an inner distance to observe or describe the situation (to take a break from reactivity)?
- How has the MB CARE program enabled you to gain the ability to focus on one thing at a time?
- How has the MB CARE program changed your relationship to time?
- How do you currently practice mindfulness?
- How many days a week and minutes a day do you use mindfulness and compassion practices?
- How much time do you use informal practices (such as a brief awareness practice)?

- How much time do you use formal practices (such as body scans or sitting meditation)?
- Where do you practice mindfulness?
- Do you ever try to explore sensations in mindfulness during your work to get through difficult times?

  Using touch (touching something comfortable),

  Using vision (looking at something beautiful),

  Using smell (smelling a scent you like),

  Using taste (eating something you like),

  Using hearing (listening to something you like),

  Affecting emotions (creating another emotion),

  Using inner images (imagining soothing scenes).
- Do you think you are more curious in your practice?

Sub-theme 2: Metacognition

Open question to address the sub-theme:

Does mindfulness have a place in your daily work today, and if so, what is it?

Follow-up questions to explore the sub-theme:

- Before you visit a patient, do you plan what you are going to say carefully or do you rather let yourself go when you are with your patient?
- When you are with your patients, do you pay attention to your surroundings and to what is going on inside you?
- What are the clues that help you to review your approach while you are treating?

- How do you detect potential conflicts? How do you deal with potential conflicts?
- Can you tell me about your ability to 'switch'?
- Can you tell me about your ability to integrate new information into your thinking and decisions?
- Can you tell me about your ability to concentrate?
- Can you tell me about your ability to manage priorities?
- To what extent do you consider that you accept your emotions and thoughts without judgment?
- Are you able to inhibit strong emotional signals?
- Are you able to regulate your emotions before you 'react'?
- Do you feel able to change your perspective on the different experiences you have in your work?
- What happens in your mind when things happen that you did not expect?
- Are you looking for an answer based on previous experience?
- Do you try to step back and let the process happen so you can understand it better?
- How do you make decisions in your practice?
- To what extent are you aware of making decisions while you are treating someone?
- What makes it easier to shift your approach when you are providing care? What makes it more difficult?
- How could you adapt your care to meet the needs of your patients?
- How do you monitor your patients when caring for them? To meet their needs for example.

  How do you monitor yourself?

  How aware are you of this monitoring?

Sub-theme 3: Empathy and compassion

Open-ended question to address the sub-theme:

Can you tell me about the relationship you have with your patients today, in terms of empathy and compassion?

Follow-up questions to explore the sub-theme:

- Do you feel you have anything in common with your patients?
- Do you feel you have a reciprocal relationship with your patients?
- Do you feel that you share a common humanity with your patients?
- Or do you feel that you are different from them or from your colleagues?
- Do you tend to criticize or blame yourself?
- Are you kind to yourself? To your patients?
- Do you sometimes feel too immersed in your emotions, that you are not present in reality?
- Do you feel you are empathetic to your patients?
  Do you think it is important to project a warm, authentic, empathetic image?
  Do you feel you have a good relationship with your patients? (therapeutic alliance)
  Can you tell me about your relationship with anxiety and concern?
  Do you feel their suffering?
  Do you ever feel bad about your patients' negative experiences?
- Do you feel that you are now able to change your perspective on your experiences, to step back?
- Do you feel that you are more accepting of the difficult experiences you encounter at work?

End of the interview

Thank you again for your participation. How do you feel after this interview?

Our aim was to get your impressions of the training in order to evaluate its impact, especially in terms of how you manage to mobilize the MB CARE method in your daily work and how it helps you in your practice.

Do you think that there are elements that we have not covered but that may be important to mention?

If you would like to receive the results of this study, I suggest you write down your email address so that we can send them to you as soon as the report is written.
